# The accuracy of an Online Sequential Extreme Learning Machine in detecting voice pathology using the Malaysian Voice Pathology Database

**DOI:** 10.1186/s40463-023-00661-6

**Published:** 2023-09-20

**Authors:** Nur Ain Nabila Za’im, Fahad Taha AL-Dhief, Mawaddah Azman, Majid Razaq Mohamed Alsemawi, Nurul Mu′azzah Abdul Latiff, Marina Mat Baki

**Affiliations:** 1 Department of Otorhinolaryngology-Head and Neck Surgery, Faculty of Medicine, Universiti Kebangsaan Malaysia (UKM), Hospital Canselor Tuanku Muhriz, Jalan Yaacob Latif, Bandar Tun Razak, 56000 Kuala Lumpur, Malaysia; 2 Faculty of Electrical Engineering, Universiti Teknologi Malaysia (UTM), 81310 Johor Bahru, Johor Malaysia; 3 College of Information Technology, Imam Ja’afar Al‐Sadiq University, Al‐Muthanna, 66001 Iraq

**Keywords:** Online Sequential Extreme Learning Machine, Accuracy, Sensitivity, Specificity, Dysphonia, Voice database

## Abstract

**Background:**

A multidimensional voice quality assessment is recommended for all patients with dysphonia, which requires a patient visit to the otolaryngology clinic. The aim of this study was to determine the accuracy of an online artificial intelligence classifier, the Online Sequential Extreme Learning Machine (OSELM), in detecting voice pathology. In this study, a Malaysian Voice Pathology Database (MVPD), which is the first Malaysian voice database, was created and tested.

**Methods:**

The study included 382 participants (252 normal voices and 130 dysphonic voices) in the proposed database MVPD. Complete data were obtained for both groups, including voice samples, laryngostroboscopy videos, and acoustic analysis. The diagnoses of patients with dysphonia were obtained. Each voice sample was anonymized using a code that was specific to each individual and stored in the MVPD. These voice samples were used to train and test the proposed OSELM algorithm. The performance of OSELM was evaluated and compared with other classifiers in terms of the accuracy, sensitivity, and specificity of detecting and differentiating dysphonic voices.

**Results:**

The accuracy, sensitivity, and specificity of OSELM in detecting normal and dysphonic voices were 90%, 98%, and 73%, respectively. The classifier differentiated between structural and non-structural vocal fold pathology with accuracy, sensitivity, and specificity of 84%, 89%, and 88%, respectively, while it differentiated between malignant and benign lesions with an accuracy, sensitivity, and specificity of 92%, 100%, and 58%, respectively. Compared to other classifiers, OSELM showed superior accuracy and sensitivity in detecting dysphonic voices, differentiating structural versus non-structural vocal fold pathology, and between malignant and benign voice pathology.

**Conclusion:**

The OSELM algorithm exhibited the highest accuracy and sensitivity compared to other classifiers in detecting voice pathology, classifying between malignant and benign lesions, and differentiating between structural and non-structural vocal pathology. Hence, it is a promising artificial intelligence that supports an online application to be used as a screening tool to encourage people to seek medical consultation early for a definitive diagnosis of voice pathology.

## Background

Dysphonia is an alteration of voice quality used by clinicians to describe any change in voice [1]. There are many causes of dysphonia, ranging from benign to malignant etiologies [2]. Examples of benign causes are benign lesions such as vocal cord polyps or nodules, inflammatory, or infective causes, laryngopharyngeal reflux, and laryngitis [2]. Premalignant lesions of the vocal folds, if left untreated, may progress to carcinoma. These vocal fold pathologies require treatment that includes a combination of medical and surgical treatments. The degree of dysphonia also varies and can be measured subjectively or objectively.

Dysphonia may affect quality of life to a certain degree, depending on the occupation and voice demand. Despite its impact on quality of life, only 6% of patients with dysphonia seek medical treatment [3] due to a lack of awareness, especially in the low-voice-demand group. Late presentation of some sinister diseases, such as laryngeal carcinoma, can lead to undesirable consequences. For example, a diagnosis of laryngeal carcinoma at an advanced stage would require a total laryngectomy.

The lifetime prevalence of dysphonia in adults less than 65 years old is 30%, with a point prevalence of 7% [4]. In Malaysia, the prevalence of dysphonia among secondary and primary school teachers is 10.4% and 53.8% respectively [5]. Professional voice users are particularly severely affected by dysphonia, contributing to work absenteeism and loss of productivity [2].

The diagnosis of voice pathology is made primarily by performing endoscopy under local or general anesthesia. Endoscopic examination using either flexible or rigid laryngoscopy is an expensive and invasive procedure. Furthermore, high-quality endoscopic imaging is not available in all centers. Multidimensional voice quality assessments are recommended to be performed in all patients with dysphonia, which includes subjective and objective assessments. Examples of subjective assessment are patient self-reported voice outcomes, such as the Voice Handicap Index-10 [6, 7] and auditory perceptual evaluation of dysphonia using dysphonia grade, roughness, breathiness, asthenia, and strain scale by a clinician [8]. Objective assessments are aerodynamic and acoustic analyses [9]. Simple aerodynamic assessments can be done by assessing the maximum phonation time [10], whereas acoustic analysis measures voice quality by feeding the recorded voices into an installed software [11]. Clinicians analyze the results of acoustic analysis according to the normal range identified for a certain population. To date, the assessment of voice pathology requires patients to visit an otolaryngology clinic.

The use of machine learning algorithms to detect voice pathology without the need to visit a physician is showing rapid development [12]. The algorithms are trained using a large dataset of voice samples of both normal and dysphonic voices and learn features that distinguish them. These features include pitch, loudness, and other acoustic characteristics. Once the algorithm is trained, it can be used to test a new voice sample and detect dysphonia based on characteristics similar to the dysphonic sample in the training set. In other words, it is used to automatically differentiate between normal and dysphonic voice [12]. The potential of machine learning algorithms to become an important tool for objective assessment of voice disorders is expected to increase with advancement in research.

Many available machine learning algorithms have proven effective and efficient in differentiating normal and dysphonic voice [12]. However, these machine learning algorithms have low execution time, with the need to retrain the entire dataset when new data are to be tested [13]. Some of these algorithms include Naive Bayes (NB) [14], Support Vector Machine (SVM) [13, 15] and Decision Trees (DT) [15], and Gaussian Mixture Model (GMM) [13].

In 2005, an Online Sequential Extreme Learning Machine (OSELM) was introduced [16]. OSELM proves to be a very fast and accurate online sequential learning algorithm and has been shown to produce higher generalization performance with less training time when compared to other machine learning algorithm [13, 16]. A recent study using the Saarbrucken Voice Database (SVD) to detect normal and abnormal voices showed an accuracy of 88% with a short execution time of 0.84 s [13].

The development of a Malaysian voice database is crucial for the accurate identification and diagnosis of voice pathology among Malaysian individuals. As different races have varying frequency perturbations [17], it is important for the database to consist of Malaysian voices to ensure accuracy in diagnosis. Although other language databases have been used in voice pathology studies, having a Malaysian voice database will provide more precise results in assessing voice disorders in this population.

In this study, the accuracy, sensitivity, and specificity of the OSELM algorithm in detecting normal and dysphonic voices using the Malaysian Voice Pathology Database (MVPD) was tested. The outcomes were compared to those of other machine learning algorithms, such as NB, SVM, and DT, to determine the most effective method for detecting voice pathology in the Malaysian population. Overall, the development of a Malaysian voice database and the use of machine learning algorithms have the potential to greatly improve the accuracy and efficiency of voice pathology detection and diagnosis.

## Methods

### Study design and study subject selection

This is a cross-sectional study that was conducted for a duration of two years in an academic tertiary laryngology clinic. The ethics board of the institution approved the study prior to data collection. Each participant testified that his/her participation was voluntary and that the decision would not affect the medical care they received.

A total of 382 participants were recruited for the study. The subjects in the study were divided into two groups: the normal voice group and the dysphonic voice group. Video laryngostroboscopy, voice recording, and acoustic analysis are routine procedures for patients with voice problems. The data for the dysphonic group were obtained from a clinic’s database of patients with voice disorders, which included video laryngostroboscopy, voice recording, acoustic analysis, and clinical diagnosis. Data that were incomplete or involved patients who had undergone laryngeal surgery or aphonic patients were excluded from the study.

Participants in the normal voice group were identified among the staff and students of Universiti Kebangsaan Malaysia and screened by using two questionnaires: the Voice Handicap Index-10 (VHI-10) [6, 7] and Reflux Symptom Index (RSI) questionnaire [18]. The inclusion criteria were a VHI-10 score of less than 7.5 [19] and RSI score of less than 13 [18] and age between 18 and 60 years old. The exclusion criteria were previous vocal fold pathology, history of smoking, history of intubation within six months, and history of upper respiratory tract infection within two weeks. Participants who met the screening criteria were further evaluated with video laryngostroboscopy, voice recording, and acoustic analysis to ensure that they were free from any vocal fold pathology. Those who exhibited normal video laryngostroboscopy, voice recording, and acoustic analysis were included in the study.

**Fig. 1:**
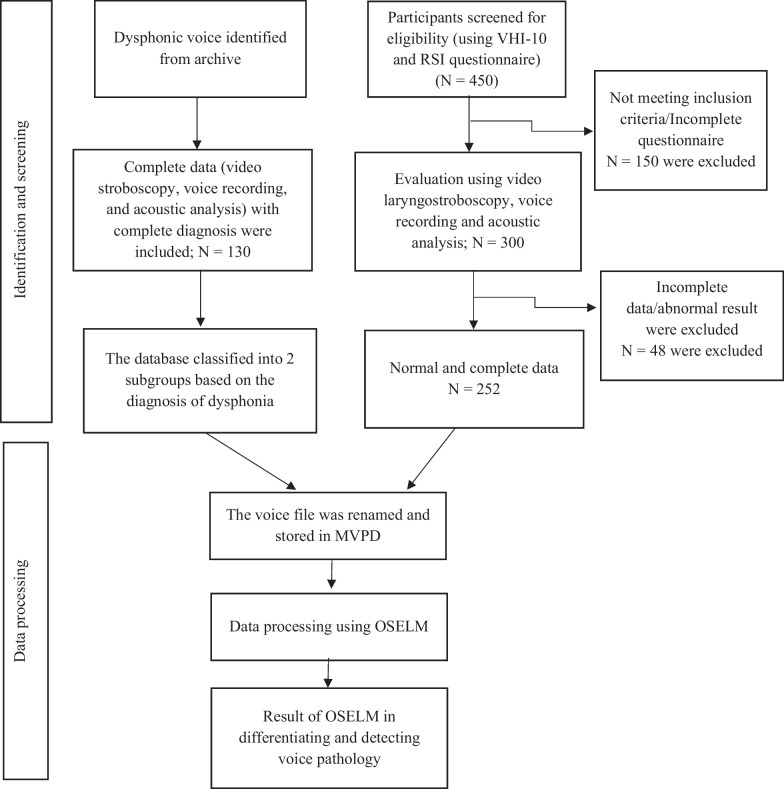
Methodology flow chart

The collected data (including video laryngostroboscopy and voice recording) were stored in a voice database named MVPD according to the two groups (normal voice and dysphonic voice). For the dysphonic voice group, the diagnoses were classified into two subgroups based on the causes of dysphonia: (1) structural, comprising malignant and premalignant, benign, and inflammatory lesions; and (2) non-structural, consisting of functional and neurogenic dysphonia. To keep the participants anonymous, the files of the collected data were assigned new names. The study methodology is summarized in Fig. 1.

### File name terminology

To ensure the confidentiality of the participants, all voice recordings were given new names with six parts. For the dysphonic group, the first part indicates the patient’s disorder, with ‘ml’ representing malignant, ‘*pm’* for premalignant, ‘*bn’* for benign, ‘*in’* for inflammatory disease, ‘*fc’* for functional, and ‘*ne’* for neurogenic. For normal subjects, the abbreviation ‘*no’* is used. The second part is a numerical code specific to each participant, while the third part denotes the participants’ age. The fourth part indicates the participant’s gender, whereby ‘*m*’ denotes male and ‘*f*’ denotes female. Next, the fifth part represents the participant’s race, using ‘*mly*’ for Malays, ‘*chi’* for Chinese, ‘*ind*’ for Indian, and ‘*oth*’ for others. The sixth part indicates the 5-s vowel /a/. For example, a voice sample named ‘in-156–28-m-ind-5a’ indicates the participant is a 28-year-old male with an inflammatory condition, and the file is the 5-s vowel /a/. All the collected voices with new names were stored in MVPD.

### Evaluation of the Malaysian Voice Pathology Database using an Online Sequential Extreme Learning Machine

**Fig. 2:**
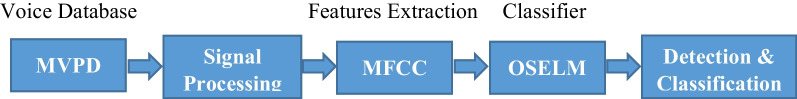
Flowchart of voice pathology detection and classification using OSELM

The voice pathology detection and classification system using the OSELM technique involves three main phases. The first phase indicates the collection of data and the creation of the proposed MVPD database. The second phase refers to the extraction of the features of voice signals. The third phase denotes the detection and classification sections. Figure 2 shows the flow of voice pathology detection and classification.

### Mel-frequency cepstral coefficient

**Fig. 3:**
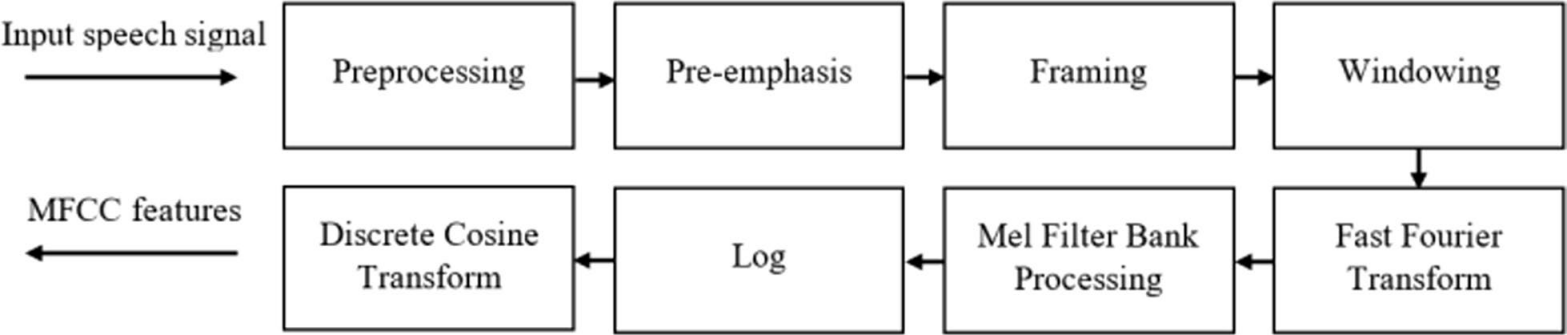
Feature extraction processes based on MFCC [13]

The Mel-Frequency Cepstral Coefficient (MFCC) technique is a tool for feature extraction in speech processing. It is widely used in automatic speech and speaker recognition systems. The process of the MFCC technique includes several steps, such as pre-emphasis, framing, windowing, Fast Fourier Transform (FFT), mel-filter bank, and Discrete Cosine Transform (DCT) [20]. The diagram of the MFCC feature extraction process is shown in Fig. 3.

In the pre-processing step, the analog signal is converted into a digital signal and the signal energy increases at a higher frequency, as in the following equation:

1 $$S_{n}^{{\prime }} = S_{n} {-}0.95*S_{{n{-}1}}$$

where *S′*_*n*_ is the new sample value, *S* is the sample value, and *n* refers to the sample number. The utterance is then separated into frames, and the Hamming window is applied to each frame. FFT is applied to each frame, with the time-domain signal converted into the frequency-domain signal. The frequency is further converted from Hertz to mel using the following equation:

2 $$f_{{mel}} = 2595 \times \log 10\left( {1 + \frac{{f_{{hz}} }}{{700}}} \right)$$

Lastly, the DCT is used to convert the log mel spectrum back into time domain. The result of conversion is called the mel-frequency cepstral coefficient [13].

### Online Sequential Extreme Learning Machine

OSELM is considered a fast algorithm, and it is able to learn from the training data through a chunk-by-chunk mechanism with constant and varying lengths. The OSELM can be used to predict an unknown input. In the OSELM algorithm, there are three layers or nodes: input layer, hidden layer, and output layer. The input layer has the extracted features, the hidden layer has biases, and the output layer has the final classes of the algorithm. The output matrix (*H*) of the hidden layer is calculated using the following equation:

3 $$H = W_{1 } \cdot X_{1 } + B_{1}$$

where *W* indicates the input weights that link the input layer to the hidden layer, *X* refers to extracted features by MFCC in the input layer, and *B* indicates biases of the hidden layer. The input weights (*W*) and hidden biases (*B*) are randomly generated with a range between − 1 and 1. For $N$ arbitrary distinct samples $\left( {{\text{x}}_{{\text{j}}} ,{\text{t}}_{{\text{j}}} } \right),{\text{where}}\quad {\text{x}}_{{\text{j}}} \in {\text{R}}^{{\text{d}}} ,{\text{and}}\,{\text{t}}_{{\text{j}}} \in {\text{R}}^{{\text{m}}}$, single layer feedforward neural networks (SLFNs) with $n$ hidden nodes and the activation function $g(x)$ can be mathematically modeled using the following equation:

4 $${{f}}({{X}}) = \mathop \sum \limits_{{i = 1}}^{n} \beta _{{\text{i}}} {\text{g}}\left( {\omega _{{{\text{I~}}}} \cdot {\text{x}}_{{{\text{j}}~}} + {\text{I}}} \right) = {\text{t}}_{{\text{j}}} ,\quad {\text{j}} = 1,2, \ldots ,{\text{N}}$$

Further, Eq. (4) can be compacted and rewritten as follows:

5 $$H \beta = T$$

where:

$$H = \left( {\begin{array}{*{20}c} {g\left( {\omega_{1} \cdot x_{1} + b_{1} } \right)} & \ldots & {g\left( {\omega_{n} \cdot x_{1} + b_{n} } \right)} \\ \vdots & \ddots & \vdots \\ {g\left( {\omega_{1} \cdot x_{N} + b_{1} } \right)} & \cdots & {g\left( {\omega_{n} \cdot x_{N} + b_{n} } \right)} \\ \end{array} } \right)_{N \times n} ,$$

$$\beta ={\left[\begin{array}{c}{\beta }_{1}^{T}\\ \vdots \\ {\beta }_{n}^{T}\end{array}\right]}_{n\times m},T={\left[\begin{array}{c}{t}_{1}^{T}\\ \vdots \\ {t}_{N}^{T}\end{array}\right]}_{N\times m}$$

The output weights ($\hat{\beta }$) is then estimated according to the following equation:

6 $$\hat{\beta } = {\text{H}}^{\dag } {\text{T}}$$

where ${H}^{\dag}$ is the Moore–Penrose generalized inverse (pseudo inverse) of the hidden layer output matrix *H*, and it is calculated as follows:

7 $${H}^{+}={\left({H}^{T}H\right)}^{-1}{H}^{T}$$

OSELM is executed to learn the training samples successively and incrementally. The learning process of OSELM consists of two steps: the initialization step and the sequential learning step. In the initialization step, the output matrix of the hidden layer ${\text{H}}_{0}$ and the output weights of the initial ${\beta}_{0}$ are calculated using the equations below:

8 $${H}_{k+1}=g\left(W\cdot {X}_{k+1}+B\right)$$

9 $${P}_{0}={\left({H}_{0}^{T}{H}_{0}\right)}^{-1}$$

10 $${\beta }_{0}={P}_{0}{H}_{0}^{T}{T}_{0}$$

In the sequential learning step, the output matrix of the hidden layer ${H}_{k+1}$ is updated for the new sample, as shown in Eq. (12). Furthermore, the output weight matrix ${\beta}_{\text{k+1}}$ is updated according to the following equations:

11 $${P}_{k+1}={P}_{k}-{P}_{k}{H}_{k+1}^{T}{\left(I+{H}_{k+1}{P}_{k}{H}_{k+1}^{T}\right)}^{-1}{H}_{k+1}{P}_{k}$$

12 $${\beta }_{k+1}={\beta }_{k}+{P}_{k+1}{H}_{k+1}^{T}\left({T}_{k+1}-{H}_{k+1}{\beta }^{k}\right)$$

**Fig. 4:**
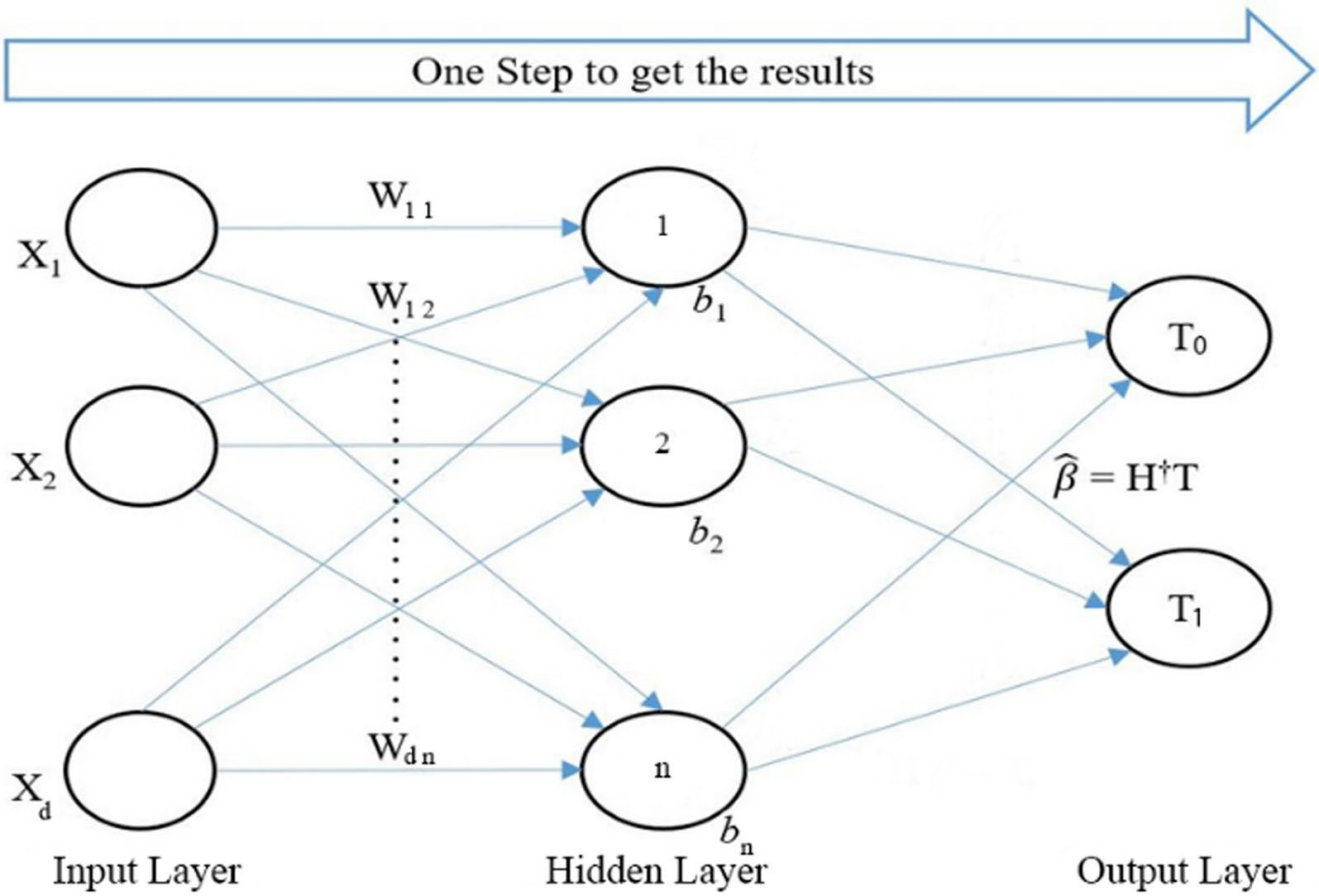
Diagram of the OSELM algorithm [13]

The set = *k* + 1 and goes back to Eqs. (8), (11), and (12) to train the next sample. When all samples are trained, the OSELM can be used to predict an unknown input vector. In the OSELM algorithm, the input layer is implemented randomly before further calculations are performed to obtain the output layer and the final results. Figure 4 shows the architecture of the OSELM algorithm, where the final classes are labeled as *T*_0_ and *T*_1_, which refer to pathological and healthy voices, respectively.

To standardize and make our results comparable with other studies, we allocated 80% of the voice samples for training the OSELM algorithm, and the remaining 20% was used for testing the OSELM algorithm [13].

## Study instruments

### VHI-10 and RSI questionnaires

The participants answered the VHI-10 and RSI questionnaires independently. Those with VHI-10 scores less than 7.5 and RSI scores less than 13 were enrolled in the normal voice group.

### Video stroboscopic examination

KayPENTAX’s laryngeal videostroboscopy system (model 9400, USA) was used in the examination, using a flexible video nasopharyngolaryngoscope and a light source. The vocal fold vibrates too fast to be seen by the naked eyes. Stroboscopy is a specialized technology that employs a special light to visualize vocal fold vibration in detail. Therefore, it can aid in the identification of vocal fold pathology.

### Voice recording and acoustic analysis

Acoustic analysis and voice recording were performed using a sixth-generation iPOD® portable media player equipped with OperaVOX™ software. The acoustic parameters measured included fundamental frequency, jitter percentage, shimmer percentage, and noise-to-harmonic ratio. The procedure was standardized to ensure the reliability of the parameters measured. Participants were asked to remove their mask while and utter vowel /a/ at a comfortable loudness for 5 s. The recording was performed in a noise-free environment. Abnormal acoustic analysis results were excluded from the study. The normative value utilized for acoustic analysis with OperaVOX ™ software was as previously described [11].

### Statistical analysis

The performance of the OSELM algorithm was evaluated using accuracy, sensitivity, and specificity. The definitions of these evaluation measurements as follows:

Accuracy: The ability of the algorithm to correctly differentiate the dysphonic voice from the normal voice. Mathematically, this can be stated as follows:

$${\text{Accuracy}} = ({\text{TP}} + {\text{TN}})/({\text{TP}} + {\text{TN}} + {\text{FP}} + {\text{FN}})$$

Sensitivity: The ability of the algorithm to correctly identify the dysphonic voice. Mathematically, this can be stated as follows:

$${\text{Sensitivity}} = {\text{TP}}/({\text{TP}} + {\text{FN}})$$

Specificity: The ability of the algorithm to correctly identify normal voice. Mathematically, this can be stated as follows:

$${\text{Specificity}} = {\text{TN}}/({\text{TN}} + {\text{FP}})$$

where, the definition of TP, TN, FP, and FN as follows:

- True positive (TP): The voice is dysphonic, and the algorithm differentiates it as dysphonic.
- True negative (TN): The voice is a normal voice, and the algorithm has identified it as normal.
- False positive (FP): The voice is normal, whereas the algorithm has identified it as dysphonic.
- False negative (FN): The voice is dysphonic, whereas the algorithm has identified it as normal.

## Results

### Demographics

A total of 382 voices were included in the study, comprising 252 (65%) with normal voice and 130 (34%) with dysphonic voice. In the normal voice group, 180 (71%) were female, and 72 (29%) were male. In the dysphonic voice group, 60 (46%) were female, and 70 (54%) were male. The age of the participants in the normal voice group ranged from 19 to 59 years old, whereas that of the dysphonic group ranged from 14 to 82 years old. With regard to ethnicities, in the normal voice group, 192 (76%) were Malay, 40 (16%) were Chinese, 17 (7%) were Indian, and 3 (1%) were other races. For the dysphonic voice group, 77 (60%) were Malays, 31 (24%) were Chinese, 18 (14%) were Indian, and 4 (3%) were other races (Table 1).

**Table 1:** Demographic distribution

Demographics	Healthy (N = 252)	Dysphonia (N = 130)
*Sex*		
Female	180 (71%)	60 (46%)
Male	72 (29%)	70 (53%)
*Age*		
Mean (years)	31.6	52.3
SD	9.5	16.4
*Ethnicity*		
Malay	192 (76%)	77 (60%)
Chinese	40 (16%)	31 (24%)
Indian	17 (7%)	18 (14%)
Others	3 (1%)	4 (3%)

The dysphonic voice group was categorized into two subgroups: structural and non-structural. The structural group consisted of those with malignant, premalignant, benign, and inflammatory lesions, while the non-structural group comprised participants with functional and neurogenic dysphonia. The malignant group, which included those with laryngeal carcinoma, and the premalignant group were grouped as ‘malignant’ during analysis, as both of these conditions require early or urgent treatment. The distribution is summarized in Table 2.

**Table 2:** Distribution of dysphonia group

Dysphonia group	Number
*Structural*	
Malignant	37
Premalignant	3
Benign	23
Vocal fold cyst	7
Vocal fold polyp	5
Vocal fold nodule	3
Laryngeal amyloidosis	3
Recurrent respiratory papillomatosis	3
Sulcus vocalis	2
Inflammatory	13
Laryngopharyngeal reflux	4
Tuberculosis laryngitis	2
Fungal laryngitis,	2
Laryngitis	2
Vocal fold edema,	2
Laryngeal perichondritis	1
*Non-structural*	
Unilateral vocal fold palsy	28
Spasmodic dysphonia	9
Bilateral vocal fold palsy	5
Voice tremor	1
Presbylarynx,	4
Primary muscle tension dysphonia	4
Puberphonia	2
Psychogenic dysphonia	1

### The accuracy, sensitivity, and specificity of OSELM and other classifiers in detecting normal and dysphonic voices

The accuracy of OSELM in detecting normal and dysphonic voices was 90%, with sensitivity and specificity of 98% and 73%, respectively. With other classifiers, the accuracy, sensitivity, and specificity in detecting normal and dysphonic voices were 80%, 70%, and 86% for NB; 75%, 75%, and 76% for SVM; and 72%, 62%, and 80% for DT (Table 3). These data showed that OSELM has superior accuracy and sensitivity in detecting dysphonic voices with a specificity comparable to other classifiers.

**Table 3:** Accuracy, sensitivity, specificity of OSELM in detecting normal voice and dysphonia and comparison with other classifiers

	OSELM (%)	Naive Bays (NB) (%)	Support Vector Machine (SVM) (%)	Decision Tree (DT) (%)
Accuracy	90	80	75	73
Sensitivity	98	70	75	62
Specificity	73	86	76	80

### The accuracy, sensitivity, and specificity of OSELM and other classifiers in differentiating structural versus non-structural vocal fold pathology

The structural vocal fold lesion group was tested for non-structural causes of dysphonia. The accuracy of OSELM in differentiating between structural and non-structural vocal fold pathology was 85%, with sensitivity and specificity of 89% and 88%, respectively. Other classifiers differentiated between structural and non-structural vocal fold pathology with accuracy, sensitivity, and specificity of 65%, 64%, and 67% for NB; 69%, 43%, and 79% for SVM; and 81%, 75%, and 83% for DT (Table 4). These results indicate the superior accuracy, sensitivity, and specificity of OSELM in identifying structural and non-structural vocal fold pathology compared to other classifiers.

**Table 4:** Accuracy, sensitivity, specificity of OSELM and other classifiers in differentiating structural and non-structural vocal fold pathology

	OSELM (%)	Naive Bays (NB) (%)	Support Vector Machine (SVM) (%)	Decision Tree (DT) (%)
Accuracy	85	65	69	81
Sensitivity	89	64	43	75
Specificity	88	67	79	83

### The accuracy, sensitivity, and specificity of OSELM and other classifiers in differentiating malignant and benign voice pathology

For the structural vocal fold pathology voices, the malignant and premalignant were grouped into malignant lesion groups to indicate the need for early or urgent treatment. The accuracy of OSELM in differentiating malignant from benign vocal fold lesions was 92%, with sensitivity and specificity of 100% and 58%, respectively. However, the accuracy, sensitivity, and specificity of differentiating malignant and benign vocal fold lesions were 67%, 50%, and 75% for NB; 62%, 53%, and 85% for SVM; and 75%, 67%, and 78% for DT, respectively (Table 5). Again, OSELM had the highest accuracy and sensitivity in classifying between malignant and benign vocal fold pathologies compared to NB, SVM, and DT. However, OSELM’s specificity was the lowest in this respect.

**Table 5:** Accuracy, sensitivity, specificity of OSELM and other classifier in differentiating malignant and benign vocal fold lesion

	OSELM (%)	Naive Bays (NB) (%)	Support Vector Machine (SVM) (%)	Decision Tree (DT) (%)
Accuracy	92	67	62	75
Sensitivity	100	50	53	67
Specificity	58	75	85	78

## Discussion

To investigate voice disorders, researchers have used voice databases of different languages to accurately detect and classify voice pathology. Some of the available databases include the Arabic Voice Pathology Database (AVPD), which was developed to evaluate voice disorders among populations in the Arab region. The Massachusetts Eye and Ear Infirmary (MEEI) was developed in the English language, and the Saarbrucken Voice Database (SVD) was developed in German [17]. Studies have shown that different ethnicities have different voice characteristics [17]. Therefore, to study voice pathology detection and classification in Malaysia, a local database was developed and tested in this study. To date, the present study is the first artificial intelligence voice pathology detection research conducted in Malaysia and the first to develop a local voice database, namely MVPD, comprising normal and dysphonic voices. Various voice pathologies were included in the database, including structural (malignant, premalignant, benign, and inflammatory lesions), neurological (vocal cord palsy, spasmodic dysphonia, and vocal tremor), and functional voice disorders.

A total of 382 voices comprising 252 (66%) normal voices and 130 (34%) dysphonic voices were included in the MVPD. The sample size in this study was determined by adapting a previous study, in which a minimum sample size of 357 participants, including 107 participants with dysphonia, was required to achieve a minimum power of 80% (actual power 81.9%) for detecting a change in the percentage value of sensitivity of a screening test from 0.80 to 0.90, based on a target significance level of 0.05 (actual *p* = 0.040) [21]. Another way of determining sample size is by balancing the number of normal and dysphonic voices [17] For example, the AVPD sample has an almost equal normal and dysphonic voice distribution [17].

We used MVPD to train and test OSELM in detecting and classifying normal and dysphonic voices. In the present study, the accuracy, sensitivity, and specificity of OSELM in (1) detecting normal versus dysphonic voices were 90%, 98%, and 73%, respectively; (2) differentiating structural versus non-structural voice pathology were 85%, 89%, and 88%, respectively; and (3) differentiating malignant versus benign voice pathology were 92%, 100%, and 58%, respectively. In comparison with other algorithms (NB, SVM, and DT), OSELM exhibited the highest accuracy and sensitivity in classifying voice pathologies. These findings showed that OSELM is a good artificial intelligence classifier in differentiating normal versus dysphonic voices, structural versus non-structural vocal folds, and malignant versus benign voice pathologies.

OSELM demonstrated superior accuracy and sensitivity in classifying voice pathology compared to NB, SVM, and DT. In terms of specificity in differentiating structural and non-structural vocal fold pathology, OSELM had the highest result; however, it had the lowest result in differentiating between malignant and benign voice pathologies. This may be attributed to overlapping voice characteristics observed in malignant and benign vocal fold lesions, as documented by a previous study, in which both malignant and benign lesions of vocal folds were shown to potentially impair the mucosal wave [22]. However, the study demonstrated a higher rate of absent mucosal waves in invasive glottic carcinoma and middle-and high-grade dysplasia compared to benign lesions [22].

The present study is comparable with a previous study that used various machine learning classifiers to classify dysphonia and normal voice using the SVD database and various classifiers [15]. Another study also showed comparable results in terms of accuracy detection and classification of vocal fold pathology using an Arabic database [17]. Compared to other machine learning classifiers, OSELM exhibits higher generalization performance and requires less training time. The OSELM algorithm supports online applications, as it does not require retraining whenever new data is received, while other classifiers require retraining for both past and new data, which can be time consuming [13, 16].

The OSELM algorithm is a promising artificial intelligence voice pathology classifier that can be used as a screening tool for detecting dysphonia. In the future, OSELM can be independently used by the general population to initially screen for structural and non-structural voice pathology. OSELM may further classify whether the structural voice pathology is malignant or benign with high accuracy and sensitivity. This would increase awareness among the general population and people can independently test their own voices remotely from the hospital. Detection of structural voice pathology would also alert the individual to seek early consultation from an otorhinolaryngology surgeon for diagnosis and treatment. This would enhance the early diagnosis of laryngeal cancer for better outcomes.

Although the proposed OSELM algorithm has been achieved promising results in the detection and classification of voice pathology with respect to the MVPD database, there are some limitations in this present study that can be summarized as follow:

1. The performance of the proposed algorithms for voice pathology detection and classification is evaluated in terms of accuracy, specificity, and sensitivity only. In other words, there are other evaluation measurements that can be used such as precision, G-mean, F-measure, and execution time.
2. The proposed machine learning algorithms have been trained and tested based on the proposed database (i.e., MVPD) using 5 s duration only. Where it is also imperative to evaluate several machine learning algorithms using different voice durations.
3. The proposed algorithms can be further trained and tested for detecting and diagnosing particular diseases of the voice box. For example, discriminate the laryngeal cancer samples from the healthy samples, as well as classify the laryngeal cancer stages.

Taking these limitations into account, we plan to address these limitations in our future work which can be summarized as follow:

1. Using many evaluation measurements to evaluate the performance of the proposed algorithms for voice pathology detection and classification.
2. Training and testing several algorithms of machine learning and deep learning based on the proposed MVPD database by using different voice durations (e.g., 1 s, 3 s, 7 s, and 10 s).
3. In our next work, we plan to use the proposed machine learning algorithms in the detection and classification of laryngeal cancer.
4. In the future, the proposed system can be used in both healthcare setting such as hospitals and clinics and by general population. In a healthcare facility which are not equipped with office setting laryngoscopes or a general practitioner who are not laryngology trained, they can use the purposed system for detection of laryngeal pathology and subsequently make appropriate medical referrals to a tertiary centre. In other words, the proposed system can be uploaded to the Cloud and the users can use their internet of things devices such as smartphones and tablets to record, capture, and upload their voices into the Cloud. Then, the voices can be processed and analyzed in the proposed system using the OSELM algorithm. Subsequently, the results will be sent back to the users to inform them about the findings with further feedback.

## Conclusion

The OSELM algorithm demonstrated high accuracy, sensitivity, and specificity of 90%, 98%, and 73%, respectively, in detecting voice pathology and the highest accuracy and sensitivity compared to other classifiers (NB, SVM, and DT). It also showed high accuracy and sensitivity in differentiating between structural versus non-structural as well as malignant versus benign voice pathology. Hence, it is a promising artificial intelligence voice pathology classifier that can be used as a screening tool to detect pathological voices and classify them.

## Data Availability

The voice database is available upon request from the corresponding author.

## Abbreviations

AVPD: Arabic Voice Pathology Database
DT: Decision Tree
DCT: Discrete Cosine Transform
FP: False positive
FN: False negative
FFT: Fast Fourier Transform
GMM: Gaussian Mixture Model
MVPD: Malaysian Voice Pathology Database
MEEI: Massachusetts Eye and Ear Infirmary
MFCC: Mel-Frequency Cepstral Coefficient
NB: Naive Bayes
OSELM: Online Sequential Extreme Learning Machine
SVM: Support Vector Machine
SVD: Saarbrucken Voice Database
TP: True positive
TN: True negative
